# A Potential Prognostic Gene Signature Associated with p53-Dependent NTRK1 Activation and Increased Survival of Neuroblastoma Patients

**DOI:** 10.3390/cancers16040722

**Published:** 2024-02-08

**Authors:** David Currie, Nicole Wong, Isabelle Zane, Tom Rix, Marios Vardakastanis, Amelia Claxton, Karine K. V. Ong, William Macmorland, Arthur Poivet, Anthony Brooks, Paola Niola, Derek Huntley, Ximena Montano

**Affiliations:** 1 Department of Life Sciences, Imperial College London, London SW7 2AZ, UK; david.currie21@imperial.ac.uk (D.C.); n.wong@inoviv.com (N.W.); iz2@sanger.ac.uk (I.Z.); tom.rix21@imperial.ac.uk (T.R.); mkvardas@gmail.com (M.V.); apoivet@genpax.co (A.P.); d.huntley@imperial.ac.uk (D.H.); 2 Innovation Hub, Comprehensive Cancer Centre, King’s College London, Great Maze Pond, London SE1 9RT, UK; amelia.claxton@kcl.ac.uk (A.C.); kai.v.ong@kcl.ac.uk (K.K.V.O.); 3 Tumour Immunology Group, School of Cancer and Pharmaceutical Sciences, King’s College London, London SE1 1UL, UK; william.macmorland@kcl.ac.uk; 4 Zayed Centre for Research into Rare Disease in Children, UCL Genomics, London WC1N 1DZ, UK; a.brooks@ucl.ac.uk; 5 Immunocore, Oxfordshire OX14 4RY, UK; paola.niola@immunocore.com; 6 School of Life Sciences, University of Westminster, London W1W 6UW, UK

**Keywords:** neuroblastoma, biomarkers, gene signature, *NTRK1*, prognosis

## Abstract

**Simple Summary:**

Neuroblastomas are the most frequent neoplasms of infancy worldwide, accounting for 8% to 10% of all childhood cancers. Long-term survival with high-risk-type tumours is poor. Given their heterogeneous biological and clinical nature, tumours display mixed cell populations with some showing spontaneous regression and others metastatic activity. There remains a lack of specific biomarkers for early diagnosis, prognosis and personalised patient-targeted therapies. Therefore, the identification of neuroblastoma biomarkers, in the form of gene signatures that distinguish high-risk from low-risk patients is essential. We have demonstrated that activation of NTRK1 by TP53-dependent repression of PTPN6 expression, is significantly associated with favourable relapse-free survival in neuroblastoma. In this investigation, we have identified and validated a seventeen-gene signature for neuroblastoma prognosis using differentially expressed genes, upon the activation of NTRK1-PTPN6-TP53. A random survival forest model was used to construct a gene signature which was then assessed across validation datasets. Results showed that the gene signature was statistically significantly associated with favourable event-free survival (EFS). Patients with higher prognostic scores had significantly lower EFS than those with lower prognostic scores. Importantly, each individual gene was significantly associated with EFS in an independent manner. Moreover, the gene-set was significantly associated with lack of *MYCN* amplification, diagnosis before 18 months of age, differentiated tumour histology and presence of NTRK1-PTPN6-TP53 activation. These results strongly suggest that the gene signature is associated with good-prognosis neuroblastomas and may have value for patient risk stratification and personalised targeted therapies.

**Abstract:**

Neuroblastoma is the most common extracranial solid tumour in children, comprising close to 10% of childhood cancer-related deaths. We have demonstrated that activation of NTRK1 by TP53 repression of PTPN6 expression is significantly associated with favourable survival in neuroblastoma. The molecular mechanisms by which this activation elicits cell molecular changes need to be determined. This is critical to identify dependable biomarkers for the early detection and prognosis of tumours, and for the development of personalised treatment. In this investigation we have identified and validated a gene signature for the prognosis of neuroblastoma using genes differentially expressed upon activation of the NTRK1-PTPN6-TP53 module. A random survival forest model was used to construct a gene signature, which was then assessed across validation datasets using Kaplan–Meier analysis and ROC curves. The analysis demonstrated that high *BASP1*, *CD9*, *DLG2*, *FNBP1*, *FRMD3*, *IL11RA*, *ISGF10*, *IQCE*, *KCNQ3*, and *TOX2,* and low *BSG/CD147*, *CCDC125*, *GABRB3*, *GNB2L1*/*RACK1 HAPLN4*, *HEBP2*, and *HSD17B12* expression was significantly associated with favourable patient event-free survival (EFS). The gene signature was associated with favourable tumour histology and NTRK1-PTPN6-TP53 module activation. Importantly, all genes were significantly associated with favourable EFS in an independent manner. Six of the signature genes, *BSG/CD147*, *GNB2L1*/*RACK1*, *TXNDC5*, *FNPB1*, *B3GAT1*, and *IGSF10*, play a role in cell differentiation. Our findings strongly suggest that the identified gene signature is a potential prognostic biomarker and therapeutic target for neuroblastoma patients and that it is associated with neuroblastoma cell differentiation through the activation of the NTRK1-PTPN6-TP53 module.

## 1. Introduction

Neuroblastoma is the most common extracranial solid tumour in children, accounting for 8% to 10% of childhood cancer-related mortality worldwide [1]. Neuroblastoma arises in the primordial neural crest cells of the sympathetic nervous system, caused by impaired cell differentiation [1]. Given the heterogenous biological and clinical nature of neuroblastoma, tumours display mixed cell populations with some showing spontaneous regression and others widespread metastatic activity regardless of therapy [2]. Due to these characteristics, the identification of neuroblastoma biomarkers in the form of gene signatures that distinguish high from low-risk patients and allow for tailored therapeutic strategies are of great importance. Key prognostic indicators have already been identified, including *MYCN* gene amplification, segmental chromosomal abnormalities (gain of chromosome 17q and hemizygous deletions of chromosomes 1p and 11q), age at diagnosis and tumour histology [3,4,5], with undifferentiated or poorly differentiated tumours associated with poor prognosis, and those with differentiated features associated with increased survival [1,6].

Neurotrophic receptor tyrosine kinase 1 (NTRK1), is the receptor for nerve growth factor (NGF) [7]. NTRK1 is activated by NGF, resulting in tyrosine (Y) phosphorylation at Y490, Y670, Y674, Y675, and Y785, which induces signalling cascades that lead to the differentiation and survival of sympathetic neurons [7]. High *NTRK1* expression is detected in favourable neuroblastomas that spontaneously regress or differentiate [8]. Research has demonstrated that the tyrosine phosphatase PTPN6 dephosphorylates NTRK1 at the residues Y670, Y674, and Y675 [9]. We have demonstrated that *PTPN6* expression is repressed by the tumour suppressor TP53, leading to NTRK1 phosphorylation at residues Y674 and Y675 in a ligand independent manner [10]. Importantly, this type of NTRK1 activation is significantly associated with 5-year relapse-free survival in neuroblastoma patients [11], strongly suggesting that TP53-dependent NTRK1 activation is an independent biomarker of good prognosis in neuroblastoma [11].

In-depth statistical analysis of datasets of non-SARS-CoV-2 infected neuroblastoma patients, to identify key genes involved with SARS-CoV-2 infection as possible neuroblastoma prognostic and infection biomarkers, has demonstrated that overexpression of *ACE2*, *CD147*, *PPIA*, and *PPIB* is significantly associated with poor-prognosis neuroblastomas [12]. This association has been observed in the presence of *MYCN amplification*, unfavourable tumour histology, and in patients older than 18 months of age [12]. Importantly, low levels of expression of *ACE2*, *CD147*, *PPIA*, and *PPIB* are significantly associated with both an active NTRK1-PTPN6-TP53 module, and good prognosis [12]. These results suggest that patients with tumours overexpressing *ACE2*, *CD147*, * PPIA*, and *PPIB* may be at a higher risk of severe SARS-CoV-2 infection [12]. Together these findings strongly suggest that *ACE2*, *CD147*, *PPIA*, and *PPIB* are potential biomarkers and therapeutic targets for neuroblastoma.

In this investigation, we hypothesised that members of a gene signature that is associated with TP53-dependent NTRK1 (NTRK1-PTPN6-TP53) activation are biomarkers for good prognosis of neuroblastoma. We have identified a gene signature from a set of 167 genes previously found to be differentially expressed by NTRK-PTPN6-TP53 activation. This gene signature consists of the 17 genes *BASP1*, *BSG/CD147*, *CCDC125*, *CD9*, *DLG2*, *FNBP1*, *FRMD3*, *GABRB3*, *GNB2L1/RACK1*, *HAPLN4*, *HEBP2*, *HSD17B12*, *IGSF10*, *IL11RA*, *IQCE*, *KCNQ3*, and *TOX2*. The genes were identified by univariate analyses to ensure each gene was individually predictive of event-free survival (EFS). A random survival forest (RSF) model was trained and validated on gene expression datasets of neuroblastoma patients. The model features included the 17-gene signature and three established prognostic markers (*MYCN* amplification, diagnosis before 18 months of age, and INSS (stages 2, 3, 4, 4S)). The resulting prognostic signature was then verified using a combination of Kaplan–Meier analysis and time-dependent receiver operating characteristic (ROC) curves. The prognostic signature was shown to be significantly associated with diagnosis at less than 18 months of age, favourable tumour histology, and lack of *MYCN* amplification on validation datasets. Gene expression was validated by quantitative PCR (qPCR) analysis. Together, our findings suggest the identification of a gene signature for potential prognosis and therapeutic target for neuroblastoma treatment.

CD147/BSG is a multifunctional transmembrane protein involved in tissue remodelling and regulation of neuronal development [13,14]. The scaffolding protein *GNB2L1*/*RACK1* is upregulated in gliomas and inhibits glioma cell differentiation [15]. *GNB2L1*/*RACK1* also promotes differentiation of intestinal epithelial cells [16]. *BASP1* is a neuronal signalling protein that is downregulated, deleted, or silenced by promoter methylation in acute and chronic lymphocytic leukaemia [17]. *FNBP1* encodes the protein FBP17, and its upregulation is linked to good prognosis of breast cancer [18]. *HEBP2* plays a role in proliferation and mitochondria-mediated cell death [19]. *CD9* encodes a tetraspanin protein whose high expression inhibits neuroblastoma development and correlates with a lack of *MYCN* amplification [20]. *DLG2* plays a role in controlling cellular proliferation and its expression is inversely correlated with *MYCN* expression [21]. Due to its location on chromosome 11q, which is deleted in aggressive neuroblastomas, *DLG2* has been considered a candidate neuroblastoma tumour suppressor gene [21].

The GABA_A_ receptor subunit *GABRB3* is expressed in the early mammalian brain and has a role in GABA_A_ receptor assembly and cell proliferation in brain development [22]. Expression of the potassium channel *KCNQ3* is associated with regulation of neuronal excitability and its downregulation in side population cells in non-small cell lung cancer has been linked to anti-cancer drug resistance [23]. *CCDC125* is a regulator of cell motility [24] and its upregulation is a marker of unfavourable prognosis in breast cancer [25]. Expression of *HAPLN4* is associated with increased motility in glioma cells [26]. IGSF10 is a protein belonging to the immunoglobulin superfamily known to regulate the migration and invasive ability of lung cancer cells [27]. *TOX2* is expressed in CD4+ T cells in classic Hodgkin lymphoma and associated with T-cell exhaustion [28]. IL11RA is a cytokine whose overexpression is associated with poor prognosis in colorectal cancer [29]. High levels of the enzyme HSD17B12 have previously been found to correlate with poor prognosis in patients with breast and ovarian tumours [30]. While the functions of *FRMD3* and *IQCE* remain unclear, *IQCE* expression is associated with improved survival of diffuse-type gastric cancer and good prognosis of endometrial cancer [31]. High expression of *FRMD3* is associated with good prognosis in non-small cell lung carcinoma but poor prognosis in rectal cancer, suggesting a tissue-specific role for this gene [32,33].

Together, our findings suggest the identification of potential gene therapeutic targets that will allow the development of neuroblastoma treatments.

## 2. Material and Methods

### 2.1. Cell Lines and Transfections

SH-SY5Y and SK-N-AS cells were maintained in RPMI media supplemented with 10% heat inactivated foetal calf serum (Gibco BRL, Waltham, MA, USA). Cells were co-transfected with pMextrk, a plasmid coding for human *proto-NTRK1*, or pLTRcG9, a plasmid coding for murine tsp53 (tsp53 Val-135) [34] and pHygro, using Lipofectamine (Gibco BRL). A total of 10^5^ cells were plated in 10 cm plates and incubated overnight with media as described above. Cells were washed 3 times with serum-free medium and DNA: Lipofectamine mix (15 μg:15 μL) was added to cells in 3 mL of serum-free Optimem medium (Gibco BRL) and left to incubate for 4 h at 37 °C, washed and incubated for another 48 h in medium supplemented with serum. Then colonies were selected in Hygromycin B containing medium (50 μg/mL) at 37 °C and resistant colonies were screened for p53 or NTRK1 expression. To obtain cells expressing tsp53 and NTRK1, clones expressing tsp53 alone were co-transfected pMextrkA and pSV2neo. Colonies were selected in G418 containing medium (100 μg/mL) at 37 °C and resistant colonies were screened for NTRK1 expression. Cell lines were maintained in 5 μg/mL Hygromycin B and G418 passaged at 70 ± 80% confluency. Cells were grown at 32 °C or 37 °C for 4 days when required. 

Neuroblastoma 9464D cells were cultured in Dulbecco’s Modified Eagle Medium (DMEM, Gibco) supplemented with 10% foetal bovine serum (FBS, Gibco), 2 mM L-Glutamine (Sigma-Aldrich, St. Louis, MI, USA), 1 mM sodium pyruvate (Hyclone, Cytvia, Marlborough, MA, USA) and 1X Non-essential Amino acids (Sigma-Aldrich). A total of 26,226 neurosphere cells were cultured in complete DMEM/F12 media (Gibco) supplemented with 15% FBS, 0.05 mM β-mercaptoethanol (Gibco), 10 ng/mL h-EGF (Peprotech, London, UK), 15 ng/mL hb-EGF (Peprotech) and 1X B27 supplement (Thermo Fisher, Waltham, MA, USA).

### 2.2. In Vivo Studies with 9464D and 262226 Cells

Seven- to fourteen-week-old C57BL/6 WT female mice were purchased from Charles River. Animal work was conducted under the Home Office project licence number PP3601022 and personal licence number I68938615. The Ethical Review Committee at King’s College London and Home Office permitted this work. The 9464D and 262226 cells were harvested for injection at 80% confluency. Cells were washed with phosphate-buffered saline (PBS, Gibco) and dissociated in PBS-based enzyme-free Cell Dissociation Buffer (Thermo Fisher) before resuspension in serum-free DMEM. Cells were centrifuged at 300× *g* for 5 min, the pellets were resuspended at 2.5 × 10^5^ per 100 µL of serum-free DMEM, for injection. Mice were anaesthetised with 3% isoflurane and injected subcutaneously with 9464D or 262226 cells in the left front flank using a 26-gauge needle. Mice were weighed weekly, and the tumours, once palpable, were measured every other day using callipers. Tumour volume was calculated using the formula (Volume = (Length*(Width2))/2), where Length refers to the longest tumour dimension. Tumours were harvested at upwards of 500 mm^3^.

Tumours were suspended in 1 mL of Invitrogen RNAlater Stabilization Solution (Thermo Fisher) in an Eppendorf tube for 24 h at 4 °C. After 24 h, the RNAlater solution was removed, and the tumour section was frozen at −80 °C for subsequent analysis.

### 2.3. RNA Extraction and cDNA Preparation

For extraction of RNA from SH-SY5Y and SK-N-AS cells, RNA was extracted using TRizol reagent (Ambion, Berlin, Germany) for RT-qPCR analysis as described by the manufacturer’s instructions. Extractions were carried out in triplicate and as experimental replicates for each cell line. RNA concentration and purity (260/280 ratio) was measured using NanoDrop (NanoDrop Technologies, Wilmington, DE, USA). RNA was retrotranscribed using the High-Capacity cDNA RT kit (Applied Biosystems, Foster City, CA, USA) and a MasterCycler Nexus (Eppendorf, Vienna, Austria), according to manufacturer’s instructions. Briefly, 2 μL buffer, 2 μL random primer, 0.8 μL dNTP mix and 1 μg total RNA was used per reaction. Sterile RNase-free water totalled the volume to 20 μL. The RT mix was incubated at 25 °C for 10 min, followed by 37 °C for 90 min and 95 °C for 5 min.

### 2.4. RNA Extraction from Murine Tumours and cDNA Preparation

A total of 30 mg of defrosted tumour tissue was weighed out. Tissue was disrupted by placing it between foil and hitting it with a hammer, then was resuspended in 600 μL buffer RLT of the RNEsay Mini Kit (Qiagen, Venlo, The Netherlands). This kit was used to complete the RNA extraction by following manufacturer’s instructions. cDNA synthesis was carried out with the same procedure as described in Section 2.3.

### 2.5. Real-Time qPCR

RT-qPCR was carried out using the QuantStudio 7 Flex Real-Time PCR System (Applied Biosystems) and conducted in a 384-well plate (Applied Biosystems) with a reaction volume of 15 μL per well. RT-qPCR was performed using SensiFAST SYBR Hi-ROX kit (Meridian) with the following gene primers for human cells: BASP1 (QT00028070), CCDC125 (QT00069881), CD147 (QT00074564), CD9 (QT00019096), DLG2 (QT00037107), FNBP1 (QT00025774), FRMD3 (QT00021721), GNB2L1 (QT01156610), HAPLN4 (QT01036056), HEBP2 (QT00073332), HSD17B12 (QT00023296), IGSF10 (QT00063329), ILL11RA (QT00035441), IQCE (QT00084091), KCNQ3 (QT00053865) and TOX2 (QT01028034). The following gene primers were used for mouse: basp1 (QT00251167), cd147 (QT00101087), cd9 (QT01752513), gnb2l1 (QT00099813), hapln4 (QT00140252), tox2 (QT01061627) and igsf10 (QT01161545). The internal controls HPRT (QT00059066) and ß-Actin (QT00016786) primers were used for human cells, whereas gapdh (QT00097146) and ß-actin (QT00118195) primers were used for mouse cells. All internal controls and gene primers were purchased from QuantiTect (Qiagen). Briefly, RT-qPCR was conducted with 7.5 μL SYBR Green PCR MasterMix, 1.5 μL primer assay, 1.5 μL cDNA and 4.5 μL RNase-free water. Each primer was tested in triplicate for each sample. Cycles (n = 40) consisted of 95 °C for 10 s and 64 °C for 30 s followed by melting curve analysis. CT values were recorded in Microsoft Excel (version 10) and analysed using 2^−∆∆CT^ method. cDNA derived from 37 °C and 32 °C cell lines were run simultaneously. Three experimental replicates were used to create an average 2^−∆∆CT^ for each gene.

### 2.6. RNA Extraction from Cell Lines and Sequencing

RNA was extracted using TRizol reagent (Ambion) from cells grown to 80% confluency. Total RNA was isolated using Total RNA Miniprep Kit (Monarch, New England Biolabs, Ipswich, MA, USA) according to manufacturer’s instructions. RNA was extracted from three independent cell populations (experimental replicates) for each cell line. RNA was quantified, sequenced and analysed using the protocols by UCL Genomics as described in the Supplementary Methods S1. Three biological replicates for each of the 6 conditions giving a total of 18 samples were sequenced. Illumina sequencing was carried out to generate paired-end reads.

### 2.7. RNA-Seq Data Analysis of Cell Lines

Salmon 0.14.1 [35] was used to assemble and map the RNA-Seq reads to the entire human transcriptome under the GENCODE reference annotation [36] (Human Release 31). After transcript quantification, tximport was used to import and prepare the counts for differential gene expression analysis with *DESeq2* [37], *edgeR* [38] and *limma-voom* [39] in BioConductor v3.19 [40] using R 3.6.0 [41]. Results were filtered with a statistically significant *p*-value cut-off of 0.05 false discovery rate (FDR) and a fold change of at least 1.5. The Benjamini–Hochberg multiple test correction procedure was used to adjust the *p*-values of all genes in the results.

The strategy to identify the differentially expressed genes (DEGs) with each package is as follows. Pairwise comparisons were conducted between the SY5Y-NTRK1-tsp53, SH-SY5Y, and SY5Y-tsp53 control cell lines grown at 32 °C or 37 °C. A *p*-value threshold of *p* < 0.05 was used to filter the DEGs. DEGs from the SY5Y-NTRK1-tsp53 list that were also detected in the SY5Y-tsp53 DEG list were filtered out unless the direction of the log1.5-fold change was different or the log1.5-fold change was in the same direction but more than 3 times higher/lower than in the SY5Y-tsp53 DEG lists. These steps were applied to the DEG lists at both temperatures using the respective cell lines, resulting in two filtered SY5Y-NTRK1-tsp53 DEG lists, at 32 °C and 37 °C. Overlapping genes in the 32 °C and 37 °C SY5Y-NTRK1-tsp53 DEG lists were removed, with only the remaining exclusive 32 °C SY5Y-NTRK1-tsp53 DEGs being kept. This process was implemented in *DESeq2*, *edgeR* and *limma-voom*. Genes identified by the 3 packages were combined to provide a comprehensive list of 167 gene predictions. These selected genes were used for further analysis. Supplementary Figure S1 shows the pipeline used to identify the DEGs. Supplementary Table S1 shows the identified 167 gene predictions.

### 2.8. Datasets

Three neuroblastoma gene expression datasets were used to train and validate the prognostic model. For training the model, GSE49711 from the Gene Expression Omnibus (GEO) database was used, consisting of 498 samples of Agilent customised 4 × 44 K oligonucleotide microarray data. This dataset was chosen for training as it is the largest of the three and has the most uniformly distributed International Neuroblastoma Staging System (INSS) values.

To validate the model, GSE85047 from the GEO database and the Paediatric Neuroblastoma study from the Therapeutically Applicable Research to Generate Effective Treatments (TARGET) initiative were used. GSE85047 contains 272 Affymetrix Exon-ST microarray samples, and the TARGET dataset contains 244 Affymetrix Exon-ST microarray samples. Clinical characteristics of each dataset are listed in Supplementary Table S2.

### 2.9. Data Pre-Processing

The model was trained and validated on patient samples that had values for event-free survival (EFS), age of diagnosis, *MYCN* amplification, and International Neuroblastoma Staging System (INSS) stage (Supplementary Table S1). The patient samples had their expression data filtered, averaged, and scaled before they were used for the models. Genes were filtered out from the expression data if they included erroneous values due to undetected reads or null values. If a gene had multiple readings in a dataset, such as from having multiple transcript-isoforms, its expression values were averaged. For the datasets that were not already Z-transformed, this scaling was performed. Patient EFS was used as the response variable for the model as relapse-free survival was not present in the datasets. EFS data was updated to have a five-year cut-off.

Only genes that were present in all three datasets were included in the model (n = 35; *B3GAT1*, *BASP1*, *BSG/CD147*, *CCDC125*, *CD9*, *CEBPD*, *CNKSR3*, *COL18A1*, *COL5A1*, *CYP3A5*, *DLG2*, *DUOX1*, *EFNA2*, *FLVCR1*, *FNBP1*, *FRAS1*, *FRMD3*, *FRMD6*, *GABRB3*, *GNB2L1*, *HAPLN4*, *HEBP2*, *HSD17B12*, *HSPA2*, *IGSF10*, *IL11RA*, *IL4R*, *INPP5F*, *IQCE*, *ISG15*, *ITGA11*, *ITGB5*, *KCNQ3*, *NEK6*, *TOX2*). To ensure that the genes would act as independent variables, pairwise Pearson correlation coefficients were calculated. The correlation coefficients for all gene pairs were less than r = 0.9. Therefore, the model should not be considerably impacted by multicollinearity.

A univariate analysis was conducted using the training data and the R package survival (v3.2-13) [42] to remove the genes that were not individually predictive of EFS. Statistical significance was measured using the Bonferroni correction (*p*-value = 0.0014 = 0.05/35), and 19 genes met this significance threshold (*B3GAT1*, *BASP1*, *BSG/CD147*, *CCDC125*, *CD9*, *DLG2*, *FNBP1*, *FRAS1*, *FRMD3*, *GABRB3*, *GNB2L1/RACK1*, *HAPLN4*, *HEBP2*, *HSD17B12*, *IGSF10*, *IL11RA*, *IQCE*, *KCNQ3*, *TOX2*)*. B3GAT1* and *FRAS1* were then removed from the list of genes as described in Section 3.2, resulting in a signature with 17 genes.

The three prognostic features used for the model were diagnosis before 18 months of age, *MYCN* amplification, and INSS (stages 2, 3, 4, 4S). Each of these features was statistically significant by univariate analysis with a Bonferroni correction (*p*-value = 0.0083 = 0.05/6).

### 2.10. Training the Model

The prognostic model was a random survival forest (RSF) model [43]. It was trained using the R package randomForestSRC (v3.1.0) [44] to predict patient EFS prognostic score using 20 features consisting of the three prognostic features and the 17-gene signature. Since a random forest model is non-parametric, it can capture the complex interactions in the modelling dataset. The model was trained with 200 trees and a depth of 3, the other parameters were set to their default value including nodesize of 15 and an mtry of 6.

### 2.11. Statistical Analysis

Validation cohorts were classified into high- and low-risk groups based on the prognostic model’s median score from the training dataset. The model was evaluated on the validation datasets by Kaplan–Meier analysis using the R package *survival* (v3.2.13) [42]. The survival rate of the groups was tested for statistical significance using the log-rank statistic with a *p*-value threshold and a Bonferroni correction of 0.0025 (=0.05/20). Additionally, the R package *SurvivalROC* (v1.0.3) [45] was used to construct time-dependent ROC curves and the area under the curve (AUC) was calculated to determine the prognostic model’s predictive ability. Supplementary Figure S2 shows a pipeline of the training and validation strategy.

To further evaluate the model, validation samples were stratified by diagnosis before 18 months of age, *MYCN* amplification, tumour histology, and NTRK1-PTPN6-TP53 module activation. The mean prognostic score of each stratified group was compared using the Mann Whitney U-test. The NTRK1-PTPN6-TP53 module was considered active in samples with TP53 and PTPN6 z-scores < 0 and NTRK1 z-scores > 0, otherwise the module was considered inactive. This approach is consistent with published studies characterising this module [11,12].

## 3. Results

### 3.1. Filtering Strategy to Identify Differentially Expressed Genes Associated with NTRK1-PTPN6-TP53 Activation

SH-SY5Y neuroblastoma cells express undetectable NTRK1 levels [46]. However, they express endogenous PTPN6 and low or undetectable levels of wild-type (WT) TP53 and non-amplified *MYCN* [46,47]. Therefore, SH-SY5Y cells co-transfected with *NTRK1* and a murine temperature-sensitive p53 (*tsp53*) [34], which expresses WT TP53 configuration in cells grown at 32 °C and promotes activation of the NTRK1-PTPN6-TP53 module were used. Cells grown at 37 °C, in which TP53 is in the mutant configuration resulting in a non-active NTRK1-PTPN6-TP53 module, were used as control. SH-SY5Y cells that only express transfected tsp53 and non-transfected cells grown at both temperatures were included as further controls.

A filtering pipeline to detect gene expression changes uniquely induced by the NTRK1-PTPN6-TP53 active module in SH-SY5Y cells was implemented by removing differentially expressed genes (DEGs) detected in cells expressing only tsp53 and non-transfected cells grown at 32 °C and 37 °C. Subsequently, only DEGs from the NTRK1-PTPN6-TP53 active module expressing cells grown at 32 °C which were not present in NTRK1-PTPN6-TP53 cells grown at 37 °C were kept as the final set of DEGs for further analysis. The filtering steps can be seen in Supplementary Figure S1. Results were filtered with a statistically significant *p*-value of 0.05 false discovery rate (FDR) and a fold change of at least 1.5. DEGs were analysed as described in Materials and Methods using *DESeq2* [37], *edgeR* [38] and *limma-voom* [39]. Genes identified by all the 3 packages were combined to provide a comprehensive list of 167 gene predictions. The list of genes can be seen in Supplementary Table S1.

### 3.2. Development of a Prognostic Model

To identify a gene signature for neuroblastoma good prognosis from the 167 genes associated with the NTRK1-PTPN6-TP53 signalling pathway, we validated the performance of the signature on neuroblastoma datasets and analysed the component genes for differentiation and cancer-related functions.

The model was developed using three datasets (GSE49711, GSE84057, TARGET). Samples were filtered to those with the required features (event-free survival (EFS), age at diagnosis, *MYCN* amplification and International Neuroblastoma Staging System (INSS) stage) and only genes present in all datasets were used (n = 35). Then a univariate analysis was performed to select for genes that are individually predictive of EFS (n = 19; *B3GAT1*, *BASP1*, *BSG/CD147*, *CCDC125*, *CD9*, *DLG2*, *FNBP1*, *FRAS1*, *FRMD3*, *GABRB3*, *GNB2L1/RACK1*, *HAPLN4*, *HEBP2*, *HSD17B12*, *IGSF10*, *IL11RA*, *IQCE*, *KCNQ3* and *TOX2*).

*B3GAT1* was removed from the signature as *B3GAT1* has a short and long isoform [48] which could have distinct functionality and cellular localisation [49]. It is not clear which isoform corresponds to the data as full information was not available. Furthermore, while the model suggested that high *B3GAT1* expression is associated with increased patient survival, previous literature suggests that cells with high levels of CD57 (the epitope synthesised by *B3GAT1*) is associated with poor prognosis, showing increased metastasis and decreased differentiation [48,49,50]. Thus, due to ambiguity in distinguishing *B3GAT1* isoforms and CD57, the gene was removed from the signature. *FRAS1* was removed from the gene signature as although the univariate analysis showed significance, Kaplan–Meier analysis demonstrated *FRAS1* with non-significant statistical association with EFS (*p*-value = 0.56). This could be attributed to the underlying statistical test that each method uses.

A univariate analysis was performed on three well known prognostic features (diagnosis under 18 months of age, presence of *MYCN* amplification, and INSS stage). Results showed that they were all statistically significant. The prognostic features were added to the list of significant genes. Therefore, the model contained 20 features (17 genes and three prognostic features) and was trained on GSE49711, then evaluated on GSE84057 and TARGET datasets.

### 3.3. The Prognostic Score Is Associated with Event-Free Survival (EFS)

**Figure 1:**
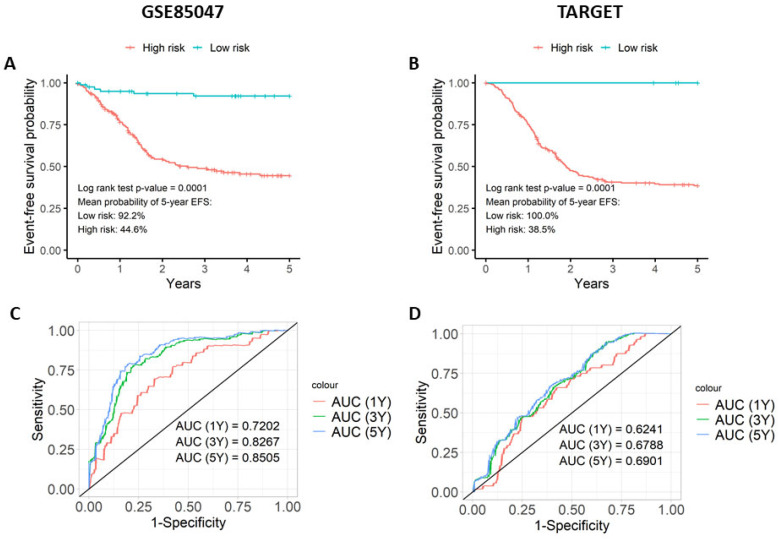
The prognostic score differentiates survival outcomes. The prognostic score was evaluated on the two validation datasets (GSE85047 and TARGET) using Kaplan–Meier plots and time-dependent ROC curves. The Kaplan–Meier plots for (**A**) GSE85047 and (**B**) TARGET compares five-year event-free survival curves for low-risk and high-risk cohorts stratified by the median risk score of GSE49711. Time-dependent ROC curve analysis for (**C**) GSE85047 and (**D**) TARGET illustrates the prognostic score’s ability to predict event-free survival in neuroblastoma patients for cohorts at one, three, and five years.

To determine if the prognostic model is predictive of EFS, the prognostic score was assessed using Kaplan–Meier and ROC curves on the validation datasets GSE85047 and TARGET (Figure 1). The Kaplan–Meier plots illustrate that patients with a higher prognostic score had lower EFS outcomes than those with lower prognostic scores (Figure 1A,B). This observation was statistically significant on both the GSE85047 and TARGET datasets (*p* < 0.0001 in both). In GSE85047 (Figure 1A), high-risk patients had a 44.6% probability of 5-year EFS with a mean survival time of 2.95 years, whereas low-risk patients had a 92.2% probability of 5-year EFS with a mean survival time of 4.68 years. Similar results were obtained with TARGET (Figure 1B). High-risk patients had 38.5% probability of 5-year EFS with a mean survival time of 3.73 years, whereas low-risk patients had 100.0% probability of 5-year EFS. Median survival times could not be computed in the two validation datasets as fewer than 50% of patients experienced an event in the two sets of risk groups.

Time-dependent ROC curves were created to measure the model’s ability to predict patients associated with EFS (Figure 1C,D). On both validation datasets, the model achieved AUC values ≥ 0.65, implying that the model can differentiate between patients that are associated with EFS and those that do not. GSE85047 gave more noteworthy results (Figure 1C) than TARGET (Figure 1D). As 87% of patients in the TARGET dataset are preferentially classified with INSS stage 4 (Supplementary Table S2), these results strongly suggest that it is a more homogenous and high-risk group of patients. Although a share of the correlation is due to the three classic prognostic features, permutation score analysis showed that the gene signature contributes to the model. The permutation scores of each gene can be seen in Supplementary Table S3.

**Figure 2:**
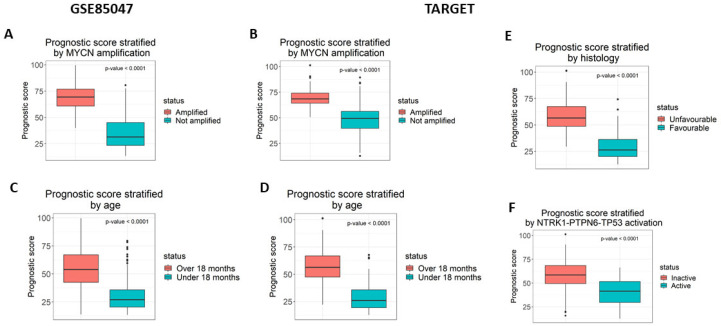
The prognostic score is significantly associated with prognostic markers. The prognostic score was measured for its association with four prognostic markers as follows: (**A**,**B**) MYCN amplification (**C**,**D**) diagnosis under the age of 18 months, (**E**) tumour histology, and (**F**) NTRK1-PTPN6-TP53 activation. The association was statistically significant for all results, using the Mann–Whitney test. GSE85047 is missing the data required to measure the association for tumour histology and NTRK1-PTPN6-TP53 activation.

The prognostic scores from the model were analysed for association with four known prognostic indicators as follows: *MYCN* amplification, diagnosis before 18 months of age, tumour histology, and NTRK1-PTPN6-TP53 module activation (Figure 2). Samples were stratified based on the presence of each prognostic indicator and the prognostic score was compared between the stratified groups using a Mann–Whitney U test with a significance threshold of *p* = 0.05. In both validation datasets, GSE85047 and TARGET, lack of *MYCN* amplification and diagnosis younger than 18 months of age was associated with a lower prognostic score (*p* < 0.0001) (Figure 2A,B). Being diagnosed before 18 months was only significant in the TARGET dataset (*p* < 0.0001) and not in GSE85047 (Figure 2C,D). GSE85047 does not include data for tumour histology nor expression data for TP53 and PTPN6, therefore these prognostic features could not be analysed for association. In the TARGET dataset, favourable tumour histology (Figure 2E) and NTRK1-PTPN6-TP53 module activation (Figure 2F) were significantly associated with a lower prognostic score (*p* < 0.0001).

### 3.4. Analysis of the Prognostic Model

**Figure 3:**
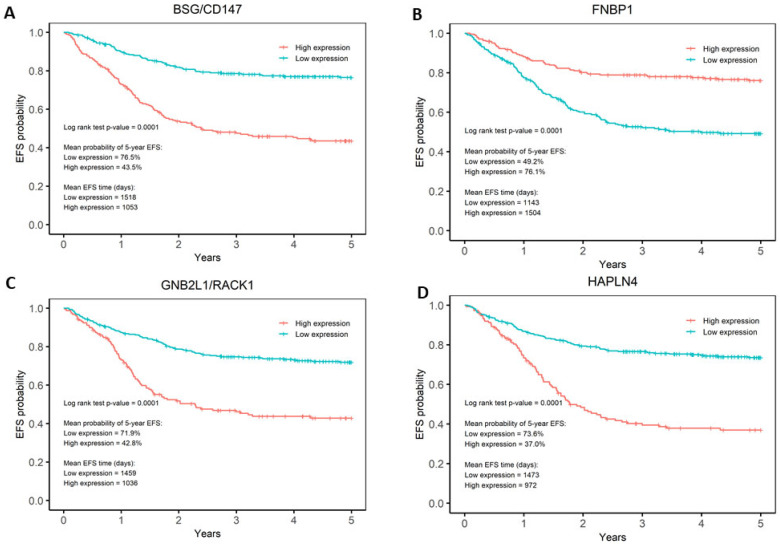
Kaplan–Meier analyses of *BSG/CD147*, *FNBP1*, *GNB2L1/RACK1*, and *HAPLN4* genes. Kaplan–Meier analysis showed that (**A**) *BSG/CD147*, (**B**) *FNBP1*, (**C**) *GNB2L1/RACK1*, and (**D**) *HAPLN4* are the genes in the signature that show the best differentiation between low- and high-risk patients. For *BSG/CD147*, *GNB2L1*, and *HAPLN4*, low expression is associated with favourable EFS. Conversely, for *FNBP1*, high expression is associated with favourable EFS.

The association between gene expression and EFS was tested separately for each gene in the prognostic signature by Kaplan–Meier analysis, to further understand the features driving the prognostic model. For each gene, EFS curves for low- and high-expression patients in GSE49711 were used in the Kaplan–Meier analysis (Supplementary Figures S3 and S4). Expression values were transformed into Z scores and defined as either low or high using 0 as the threshold. All genes were significantly associated with EFS after Bonferroni correction (*p* < 0.0026 = 0.05/19). The analysis identified that high expression of *BASP1*, *CD9*, *DLG2*, *FNBP1*, *FRMD3*, *IL11RA*, *ISGF10*, *IQCE*, *KCNQ3*, and *TOX2* and low expression of *BSG/CD147*, *CCDC125*, *GABRB3*, *GNB2L1/RACK1*, *HAPLN4*, *HEBP2*, and *HSD17B12* was significantly associated with favourable EFS (Figure 3, Supplementary Figures S3 and S4). Hazard ratios were calculated for each gene using a univariate analysis on the GSE49711 dataset to quantify the relative risk of each expression for every gene. Figure 3 highlights the most performant genes in the signature. *BSG/CD147*, *FNBP1*, *GNB2L1/RACK1*, and *HAPLN4* are the genes that can best differentiate between low-risk and high-risk patients. Patients with low gene expression of *BSG/CD147*, *CCDC125*, *GABRB3*, *GNB2L1/RACK1*, *HAPLN4*, *HEBP2*, and *HSD17B12* have a mean probability of 5-year EFS of >70%, while patients with high expression of *BASP1*, *CD9*, *DLG2*, *FNBP1*, *FRMD3*, *IL11RA*, *ISGF10*, *IQCE*, *KCNQ3*, and *TOX2* have a mean probability of 5-year EFS of <50% (Figure 3, Supplementary Figures S3 and S4).

### 3.5. RT-qPCR Analysis of the Gene Signature in Neuroblastoma

**Figure 4:**
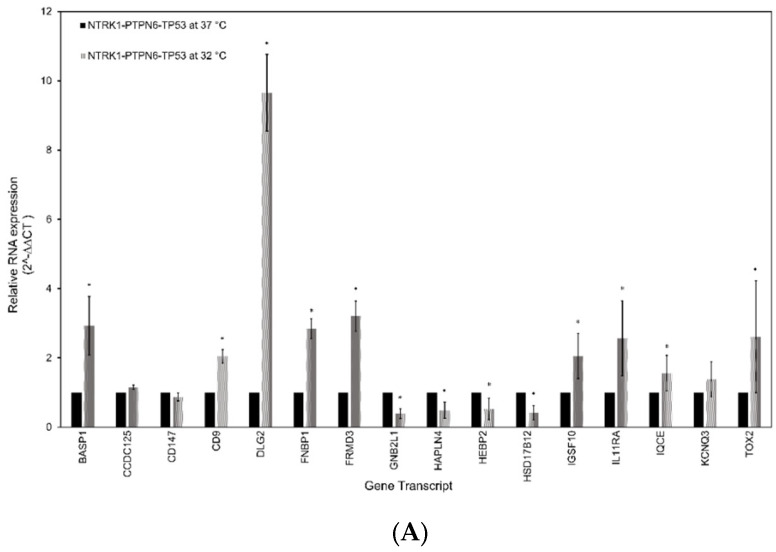

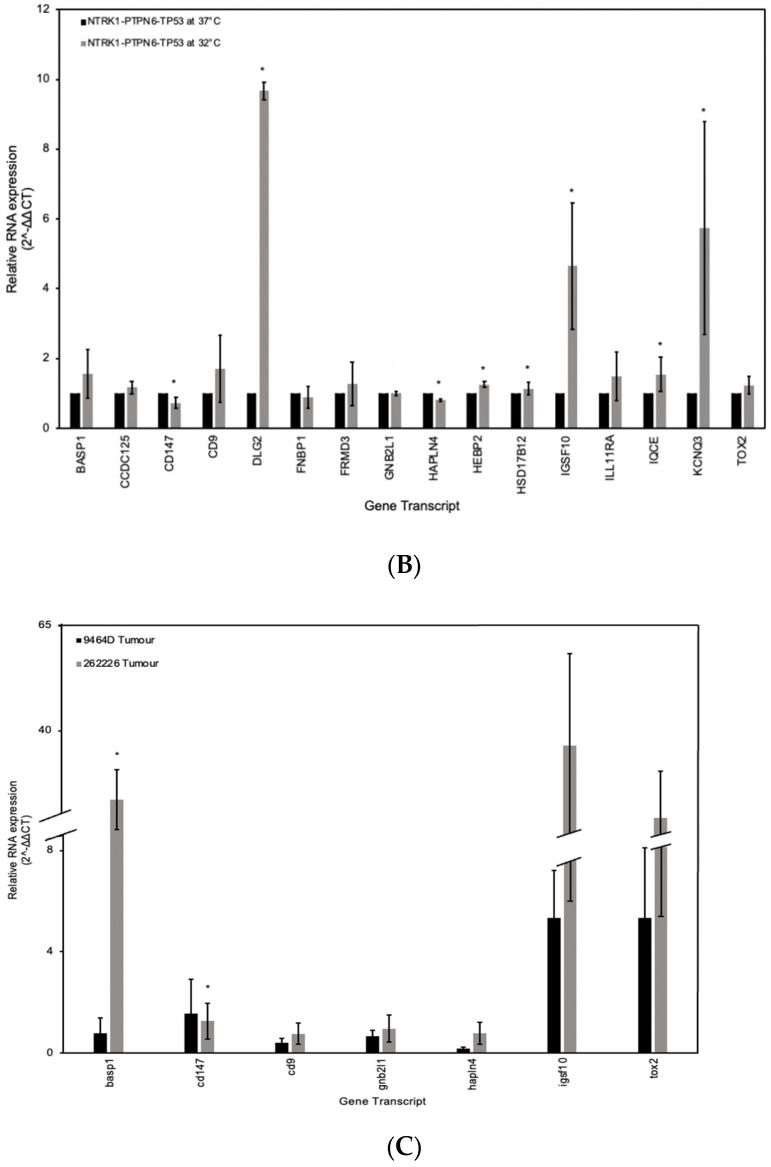
Gene signature expression validation by RTqPCR. RT-qPCR analysis of the sixteen-gene signature in human neuroblastoma (**A**) SH-SY5Y and (**B**) SK-N-AS cell lines, both expressing the NTRK1-PTPN6-TP53 module at 32 °C and lacking the expression of the module at 37 °C. The key genes from the sixteen-gene signature were also analysed in neuroblastoma murine xenografts (**C**) 9464D and 262226. Relative mRNA abundance was determined by the 2−∆∆CT method. Statistical significance was denoted as * = *p* < 0.05 (paired *t*-test). Error bars represent the standard error of the mean calculated from 2 or 3 independent experimental replicates.

The gene signature was validated using RT-qPCR. RNA extracted from SH-SY5Y and SK-N-AS cells grown at 32 °C and with an activated NTRK1-PTPN6-TP53 module was compared to RNA extracted from NTRK1-PTPN6-TP53 cells grown at 37 °C and without the activated module. As controls, RNA from SH-SY5Y and SK-N-AS non-transfected and tsp53 transfected cells grown at 32 °C or 37 °C were used. The relative mRNA abundance of each gene was obtained by the 2^−∆∆CT^ method, normalised against controls β-actin and HPRT mRNA. SH-SY5Y cells with an activated NTRK1-PTPN6-TP53 module demonstrated a statistically significantly greater abundance of mRNA transcripts corresponding to genes *BASP1*, *CD9*, *DLG2*, *FNBP1*, *FRMD3*, *IGSF10*, *IL11RA*, *IQCE,* and *TOX2* (*p* = < 0.05) (Figure 4A). Additionally, mRNA transcripts corresponding to genes *GNB2L1/RACK1, HAPLN4, HEBP2* and *HSD17B12* had a significantly lower abundance in cells with an active NTRK1-PTPN6-TP53 module, compared to cells lacking the activated module (*p* = < 0.05). *CCDC125, BSG/CD147* and *KCNQ3* did not reach statistical significance. However, *CCDC125* and *KCNQ3* mRNA abundance was greater in cells with activated NTRK1-PTPN6-TP53 (2^−∆∆CT^ 1.154, *p* = 0.17, 2^−∆∆CT^ 1.379, *p* = 0.282, respectively), whilst *BSG/CD147* mRNA abundance was lower in cells with an activated NTRK1-PTPN6-TP53 module (2^−∆∆CT^ 0.872, *p* = 0.075), ultimately following the expected trend. Similar results were observed with SK-N-AS cells. Cells with an activated NTRK1-PTPN6-TP53 module demonstrated a statistically significantly greater abundance of mRNA transcripts corresponding to genes: *DLG2, IGSF10, IQCE* and *KCNQ3* (*p* = < 0.05) (Figure 4B). Although *BASP1, CD9, FRMD3, IL11RA* and *TOX2* did not reach statistical significance, their mRNA abundance was greater in cells with an activated NTRK1-PTPN6-TP53 (2^−∆∆CT^ 1.563, *p* = 0.667, 2^−∆∆CT^ 1.710, *p* = 0.540, 2^−∆∆CT^ 1.279, *p* = 0.781, 2^−∆∆CT^ 1.488, *p* = 0.397 and 2^−∆∆CT^ 1.232, *p* = 0.140) (Figure 4B). *CD147* and *HAPLN4* showed a statistically significantly lower mRNA abundance (*p* = < 0.05). For *CCDC15* and *FNBP1*, the expectation was an increase in expression, and for *GNB2L1, HEBP2* and *HSD17B12* was a decrease in mRNA abundance. SH-SY5Y and SK-N-AS cells express wt-TP53 and the β isoform of TP53, respectively. Although both cell lines do not express endogenous NTRK1 and amplified MYCN [46,47,51,52], we cannot exclude the possibility that the differences in the transcriptional background of these cells could impact on the expression of these genes.

The expression of members of the signature was also assessed using mRNA of murine xenografts. The 9464D and 262226 cell lines which express amplified *MYCN* and mutant and WT TP53, respectively [53,54], were used to generate murine xenografts in C57BL/6 WT mice. Tumour histology was verified, and sections were stained with Ki-67 to assess proliferation index (Supplementary Figure S5). The relative mRNA abundance of each gene was obtained using the 2^−∆∆CT^ method, normalised against controls β-actin and GAPDH mRNA analysis of mRNA expression abundance of *basp1, cdc147, cd9, gnb2l1/rack1, hpln4, igsf10,* and *tox2* genes showed statistically significant high expression of *basp1* (*p* = < 0.05), and an increase of *cd9, gnb2l1/rack, igsf10 and tox2* mRNA abundance was observed (2^−∆∆CT^ 0.764, *p* = 0.773, 2^−∆∆CT^ 0.965, *p* = 0.779, 2^−∆∆CT^ 0.36.568, *p* = 0.427, 2^−∆∆CT^ 19.402, *p* = 0.606). Moreover, a statistically significant decrease in expression of *cd147* was seen as expected (*p* = < 0.05). Expression of *hapln4* shows a small increase, which could be due to the murine background of the cells.

Overall, the RT-qPCR analysis was able to validate the bioinformatically determined gene signature, with upregulated and downregulated mRNA transcripts correlating with activation of the NTRK1-PTPN6-TP53 module. For the analysis of human gene differential expression, *GABRB3* was not accessed due to non-specific activity of the primers. Since we aimed to have a comparable expression analysis with the murine RT-qPCR, not all genes in the signature were assessed due to lack of murine primer availability by the manufacturer and specificity.

## 4. Discussion

Neuroblastomas have a high heterogeneous clinical presentation. Survival outcomes severely differ between high- and low-risk neuroblastoma, presenting a critical need for reliable prognostic biomarkers to predict patient outcome. Following our observations that TP53-dependent activation of NTRK1 is statistically significantly associated with favourable neuroblastoma patient survival [11], we hypothesised that an activated NTRK1-PTPN6-TP53 module could be an independent predictor of higher survival probability of neuroblastoma patients. A total of 167 genes differentially expressed by TP53-dependent NTRK1 activation were first measured for their statistical significance by a univariate analysis. The gene signature of 17 significant genes and three prognostic markers (diagnosis under 18 months of age, presence of *MYCN* amplification, and INSS stage) were then input into an RSF model that could significantly differentiate EFS of patients from publicly available datasets. The predictive ability of the signature was able to stratify patients by other prognostic features such as *MYCN* amplification, age at diagnosis, and histology, suggesting that the signature is an independent predictor of survival outcome. Other than *GABRB3*, the expression pattern in human cell lines SH-SY5Y and SK-N-AS was experimentally validated under TP53-dependent NTRK1 activation by RT-qPCR analysis of all genes in the signature. Given that both cell lines have different molecular backgrounds, in SK-N-AS cells, an increase in *FNBP1* expression and a decrease of *CCDC15, GNB2L1, HEBP2,* and *HSD17B12* was not observed. Although SK-N-AS cells do not express WT TP53, they express the p53β isoform which is an isoform that modulates transcriptional WT TP53 activity [55,56]. Moreover, data have suggested that p53 isoforms can be regulated in a TP53 dependent and independent manner [57]. Therefore, we cannot exclude the possibility that p53β could impact on SK-N-AS cells transcriptional machinery. The selection of genes belonging to the signature were assessed in murine xenografts, we were able to assess only a selection of genes due to primer unavailability. Our results show that upregulation of *basp1, cd9, igsf10* and *tox2* and downregulation of *cd147* validates the results obtained with the human cell lines. Validation was not possible in the case of *gnb2l1* and *hpalnl4*. This is most likely due to differences in expression between human and mouse. Differences in gene expression between human and murine models are well documented [58,59]. Overall, results obtained by the RT-qPCR have been able to validate the results of our model.

The upregulation of genes *BSG/CD147*, *CCDC125*, *GABRB3*, *GNB2L1/RACK1*, *HAPLN4*, *HEBP2*, and *HSD17B12* is associated with poor neuroblastoma prognosis and low EFS. Of these, *BSG/CD147*, *HEBP2*, *HSD17B12*, and *GNB2L1/RACK1* have well characterised functions in cancer and cell survival. High expression of *BSG/CD147* is a marker of poor prognosis in head and neck squamous cell carcinoma, breast and ovarian cancer [60,61,62]. *BSG/CD147* has been detected in neuroblastoma-derived exosomes, suggesting it could be regulating the tumour microenvironment to promote neuroblastoma invasion, angiogenesis and metastasis [63]. Its inhibition has been tested in cell lines for the treatment of malignant melanomas with promising results [64,65], and therefore *BSG/CD147* inhibition could be investigated in neuroblastomas. Furthermore, the critical role of *BSG/CD147* for SARS-CoV-2 infection [66], suggests that drugs used for preventing *BSG/CD147* mediated viral entry could be repositioned for neuroblastoma treatment. *HEBP2* upregulation is a biomarker of poor prognosis in breast and lung cancer, likely a result of dysregulated microtubule dynamics during mitosis [67]. Additionally, *HEBP2* is known to promote mitochondria-mediated cell death [19], and its upregulation could result in dysregulated cell survival [19]. Both functions could be relevant to the development of neuroblastoma. *HSD17B12* is involved in arachidonic acid synthesis, whose downstream metabolites promote tumorigenesis [68]. Accordingly, *HSD17B12* upregulation is associated with poor prognosis of ovarian cancer [69]. Therefore, it could be involved with neuroblastoma poor prognosis through a similar unknown pathway. *GNB2L1/RACK1* promotes cell migration and proliferation in neuroblastomas and gliomas. Moreover, in gliomas it was observed to inhibit differentiation [15,70].

Other genes which our results show upregulation and association with poor neuroblastoma prognosis have functions associated with nervous system development, though not of neuroblasts. The role of *CCDC125* in neuroblastoma remains uncharacterised, but its upregulation is a good prognostic biomarker for gastric and thyroid cancers, which contrasts to our prediction in neuroblastoma [24,71], suggesting possible tissue-specificity. It delays cell motility and dysregulates RhoGTPase pathways to cause impaired motor neuron function in Isaac’s Syndrome [72]. Together these findings suggest that *CCDC125* may be a potential target for further neurophysiology studies and could be implicated in early neurodevelopment and neuroblastoma. *GABRB3* mutation is associated with epilepsy and autism, highlighting a function in embryonic development that could implicate its role in developing neuroblasts [73]. *HAPLN4* regulates assembly of the perineural network in adult brains [74]. Therefore, its upregulation could lead to compromised cell motility and adhesion. These genes suggest a neurodevelopmental and neurophysiological role captured by the gene signature.

The upregulation of genes *BASP1*, *CD9*, *DLG2*, *FNBP1*, *FRMD3*, *IGSF10*, *IL11RA*, *IQCE*, *KCNQ3*, and *TOX2* are associated with favourable neuroblastoma EFS. *CD9* upregulation inhibits neuroblastoma metastasis, whilst also associated with favourable prognosis and lack of *MYCN* amplification [20]. *DLG2* expression is negatively correlated with *MYCN* amplification and its location on chromosome 11q, a common chromosomal deletion in unfavourable neuroblastomas, further validates that it is a potential neuroblastoma prognostic biomarker [21].

*BASP1*, *IQCE* and *KCNQ3* are associated with differentiation and development. Through interactions with WT1, a key transcription factor implicated in Wilms’ tumour, *BASP1* and *WT1* promote tumour-suppressing gene expression, induce cell differentiation, and upregulate genes for neurite outgrowth [75]. *IQCE* promotes Hedgehog signalling, a critical pathway for normal tissue development and morphogenesis [76]. *IQCE* deletion can lead to abnormal cilia and limb development [77,78]. *KCNQ3* gain-of-function mutations are associated with neurodevelopmental disorders such as severe paediatric epilepsy and autism [79]. *KCNQ3* plays a similar role as the GABA_A_ receptor in neurotransmission during early development [80], emphasising the key role of neurodevelopmental genes and suggesting an association with neuroblastoma. Interestingly, inhibition of *KCNQ3* expression in PC12 cells results in promotion of NGF-dependent neurite outgrowth [81], as our signature suggests that upregulation of *KCNQ3* may promote NGF-independent differentiation, the role of *KCNQ3* in the context of NGF-dependent and -independent NTRK1 activation needs to be analysed further.

*FNBP1*, *FRMD3*, *IGSF10*, *IL11RA*, and *TOX2* have known roles in cancer. *FNBP1* upregulation appears to be associated with invasive tumours in breast, gastric, and bladder cancers [82]. However, a study analysing tumours without specified invasiveness observed that *FNBP1* was upregulated in favourable prognosis of cancers including breast, lung, cervical and bladder cancer [18]. It was suggested that this inconsistency could be related to the specific phenotype of invasiveness [18]. Therefore, *FNBP1* could be analysed on different neuroblastoma stages. *FRMD3* is a tumour-suppressor in non-small cell lung carcinoma as it induces apoptosis [32]. However, in rectal and colon cancers its upregulation is associated with poor prognosis, metastasis, and poor chemoradiotherapy response [33,83], suggesting its activity is tissue-specific. *IGSF10* expression is significantly decreased in lung cancer patients [27]. It is also upregulated in osteoblast differentiation, suggesting an association with early embryo development pathways which may correspond to neuroblastoma [84]. Previous studies of *IL11RA* suggest that it has a tumorigenic effect in gastric, endometrial, colorectal, and breast cancer by suppressing inflammation and promoting proliferation [85]. *IL11RA* also promotes differentiation pathways in haematopoiesis and osteoblasts/osteoclasts [85]. Therefore, *IL11RA* may promote differentiation in neuroblastoma. In breast cancer cell lines, *IL11RA* overexpression induces reduction of cell proliferation [86], which supports good prognosis. Given the numerous pathways activated by IL11 signalling (the ligand of *IL11RA*), including JAK-STAT, Ras-MAPK, PI3K-AKT, and NF-κB [87,88], the complexity of signalling and interaction could mean tissue- and pathway-specificity for the effect of IL11RA upregulation on neuroblastoma. *TOX2* upregulation is associated with decreased overall survival in NK/T-cell lymphomas [89] and progression of colorectal cancer [90], which contrasts with the signature-predicted effect for neuroblastoma. However, it is upregulated during NK cell differentiation and cell proliferation [91]. Therefore, it may promote neuroblastoma differentiation, but these tissue-specific effects need to be studied further.

## 5. Conclusions

Together our results strongly suggest that this investigation has identified a potential gene signature which is statistically significantly associated with good prognosis of neuroblastoma. Furthermore, this signature has potential to be a target for treatment and a strong indicator of gene expression changes underlying NGF independent NTRK1 activation promoting differentiation of neuroblastoma cells.

## Data Availability

All data used for this research are publicly available and presented in the Supplementary Materials.

## Supplementary Materials

The following supporting information can be downloaded at: https://www.mdpi.com/article/10.3390/cancers16040722/s1, Figure S1: Pipeline used to identify differentially expressed genes. Figure S2: Pipeline used for the training and validation of the gene signature. Figure S3: Kaplan-Meier analyses of BASP1, CD9, DLG2, FRMD3, IL11RA, IGSF10, IQCE, KCNQ3 and TOX2. Figure S4: Kaplan-Meier analyses of CCDC125, GABRB3, HEBP2, and HSD17B12. Figure S5: Histology and immunohistochemistry of murine xenografts neuroblastoma tumours. Table S1: List of 167 genes identified through analysis with Dseq2, EdgeR and limma-voom software packages. Table S2: Clinical characteristics in data sets. Table S3: Independent permutation scores of genes showing their contribution to the model.
