# Supplementary material for: A Potential Prognostic Gene Signature Associated with p53-Dependent NTRK1 Activation and Increased Survival of Neuroblastoma Patients
Source: Cancers (Basel). 2024 Feb 8;16(4):722. doi: 10.3390/cancers16040722 (PMC10886603; doi:10.3390/cancers16040722)
Supplement: Supplementary file 1 [file cancers-16-00722-s001.zip › cancers-2821578 - Supplementary Figure .pdf]

Supplementary Figure S1

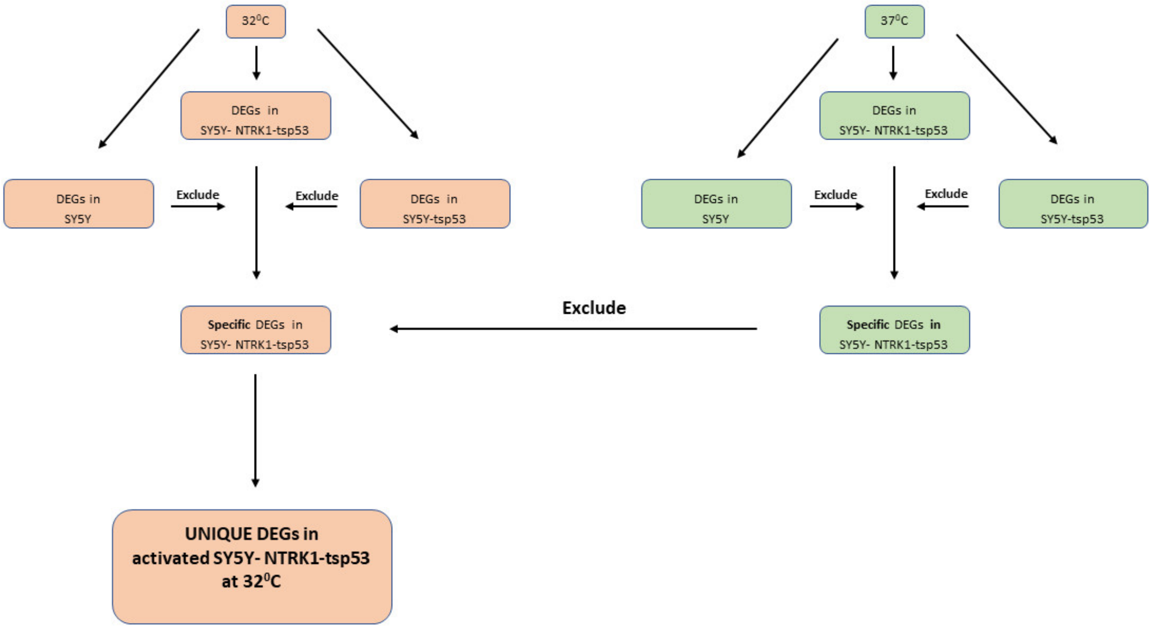

Figure S1. Pipeline used to identify differentially expressed genes. SY5Y-NTRK1-tsp53, SY5Y, and SY5Y-tsp53 cell lines were grown at 32°C (left, orange) and 37°C (right, green) with the latter two serving as control cell lines. Differentially expressed genes were identified using DESeq2, edgeR, and limma-voom, and were filtered for those that were detected exclusively in the SY5Y-NTRK1-tsp53 cell line grown under 32°C to form the final list of genes.

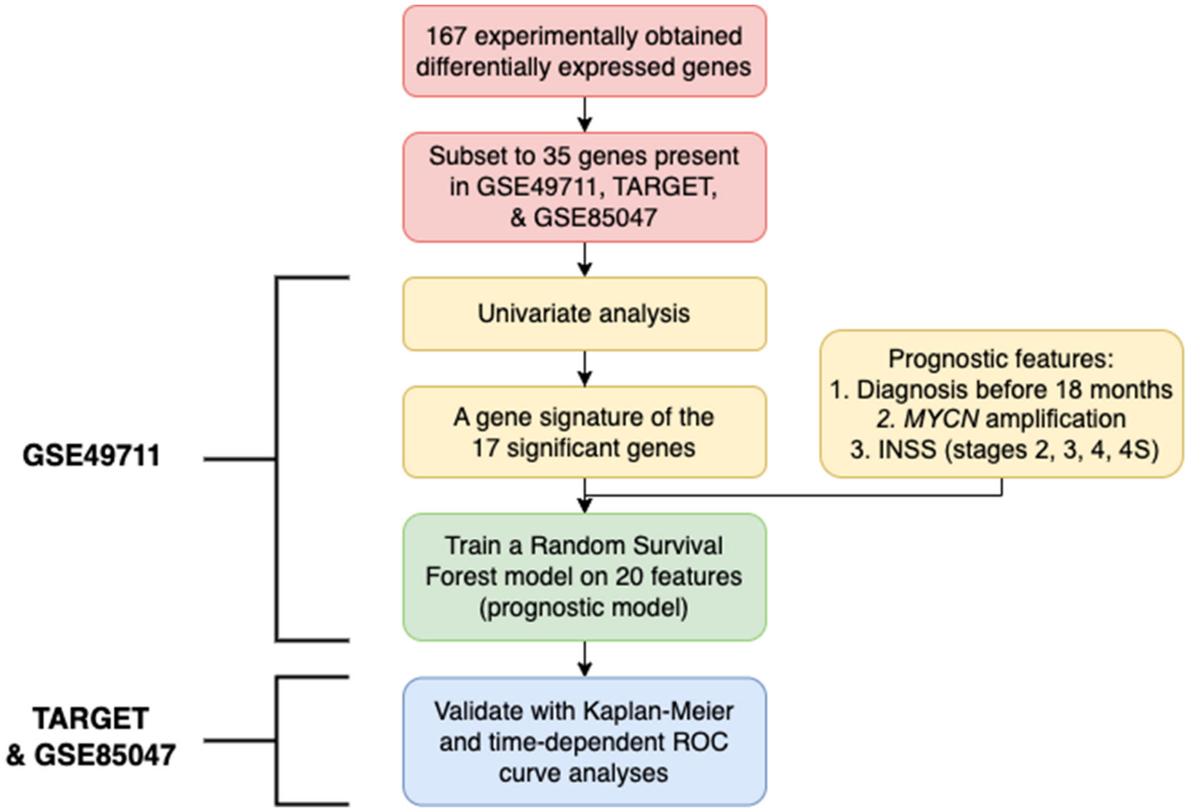

Figure S2. Pipeline used for the training and validation of the gene signature.

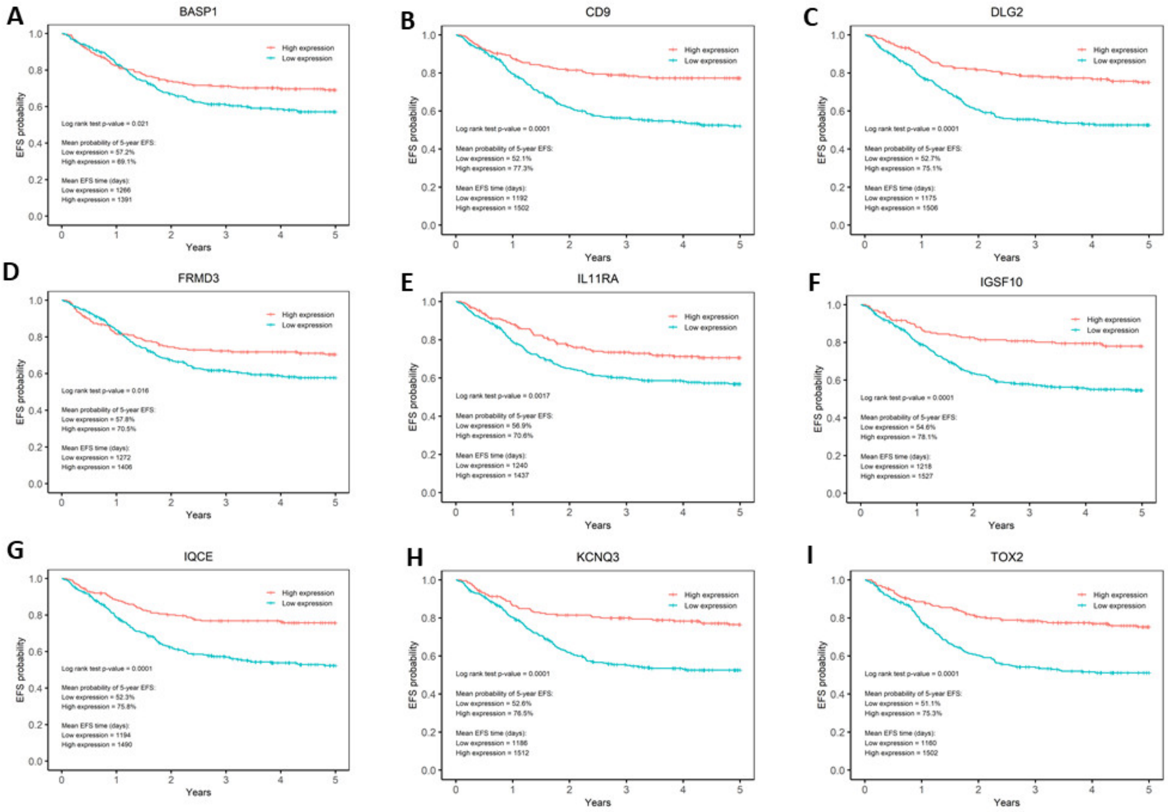

Figure S3. Kaplan-Meier analyses of *BASP1*, *CD9*, *DLG2*, *FRMD3*, *IL11RA*, *IGSF10*, *IQCE*, *KCNQ3* and *TOX2*. Kaplan-Meier analysis showed that high expression of the genes **A**) BASP1, **B**) *CD9*, **C**) *DLG2*, **D**) *FRMD3*, **E**) *IL11RA*, **F**) *IGSF10*, **G**) *IQCE*, **H**) *KCNQ3*, **I**) *TOX2* defined as Z-score > 0 in the dataset GSE49711, is significantly associated with favourable EFS after Bonferroni correction ($p < 0.0026$).

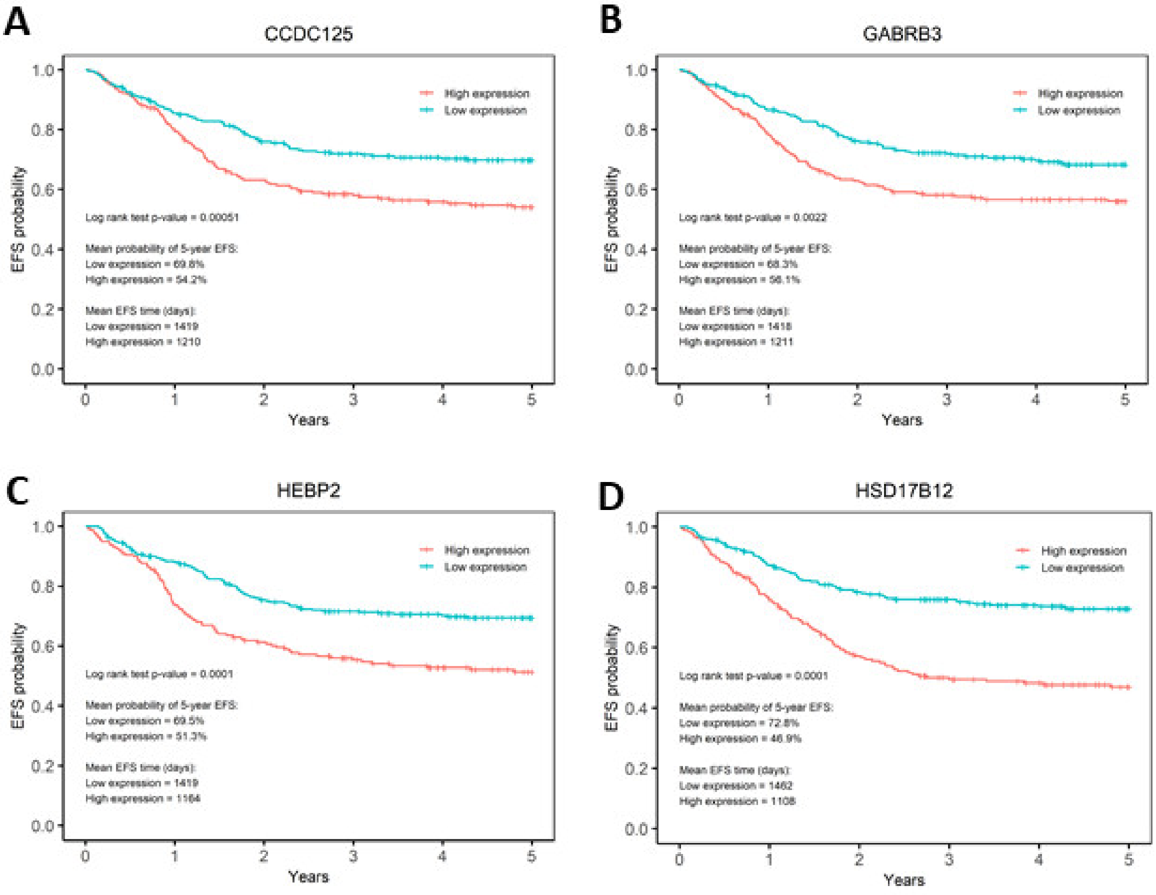

Figure S4. Kaplan-Meier analyses of *CCDC125*, *GABRB3*, *HEBP2*, and *HSD17B12*. Kaplan-Meier analysis showed that low expression of the genes **A**) *CCDC125*, **B**), *GABRB3*, **C**) *HEBP2*, and **D**) *HSD17B12*, defined as Z-score < 0 in the dataset GSE49711, is significantly associated with favourable EFS after Bonferroni correction ($p < 0.0026$).

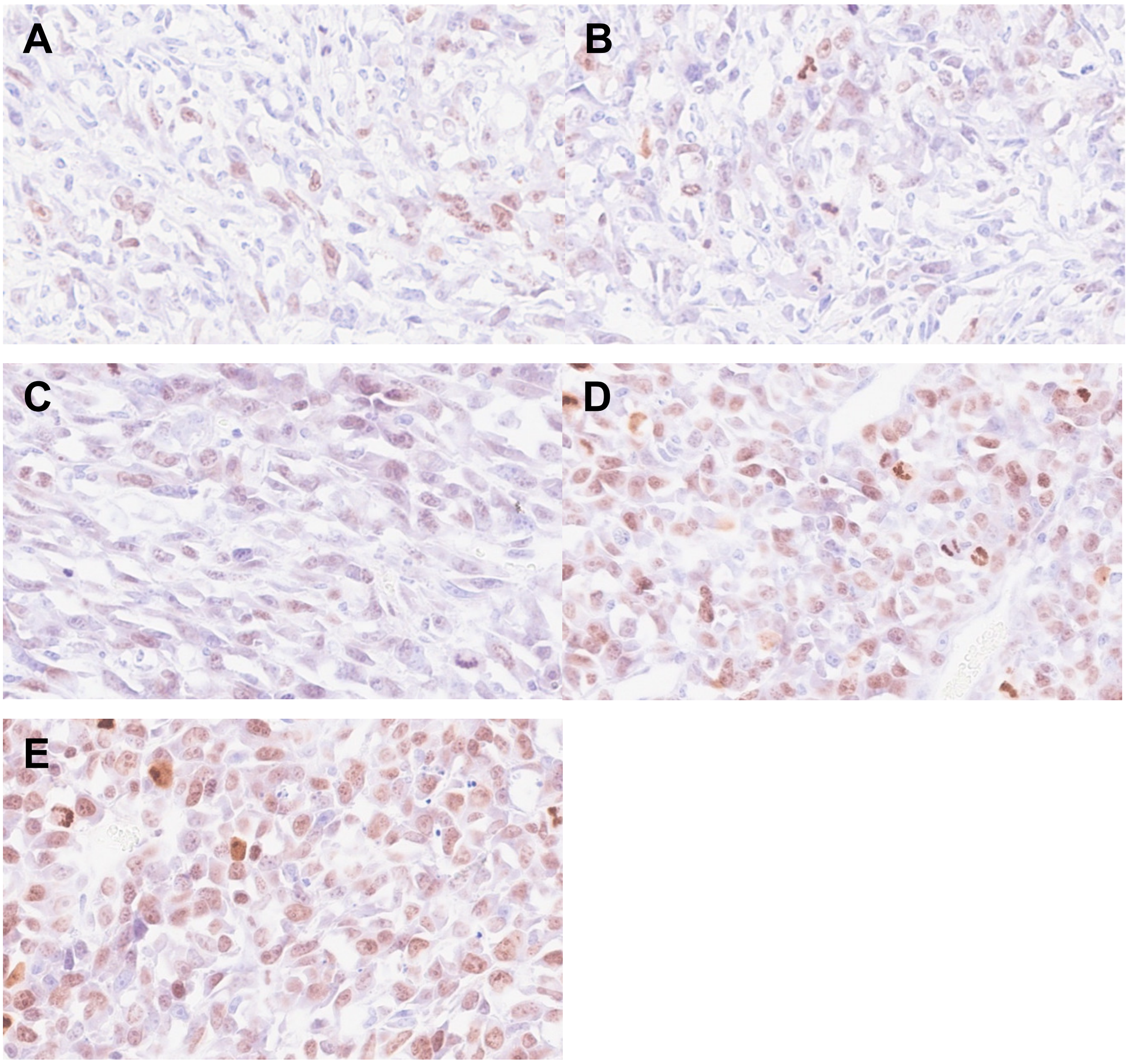

Figure S5. Histology and immunohistochemistry of murine xenografts neuroblastoma tumours. Close to 1 gram of tumour tissue was placed 3 ml of 4% paraformaldehyde (Sigma Aldrich) and left to incubate overnight at 4^{0}C. The formalin was removed and replaced with 30% sucrose for overnight incubation at 4^{0}C. Subsequently the tissue section was embedded in OCT mounting media (VWR) and snap freeze on dry ice. sections were cut (4 microns) and staining was carried out using rabbit anti-mouse Ki-67 antibodies (Abcam) using the ImmPRESS HRP goat anti-rabbit IgG polymer kit peroxidase (Vector Laboratories) and the DAB substrate chromogen system (DAKO). Expression of Ki-67 staining in **(A** and **B)** 262226 and **(C** to **E)** 9464D individual tumours. The Ki-67 proliferation index was graded as positive or negative. All fields of view were imaged at x40 magnification.
