# Supplementary material for: A Potential Prognostic Gene Signature Associated with p53-Dependent NTRK1 Activation and Increased Survival of Neuroblastoma Patients
Source: Cancers (Basel). 2024 Feb 8;16(4):722. doi: 10.3390/cancers16040722 (PMC10886603; doi:10.3390/cancers16040722)
Supplement: Supplementary file 1 [file cancers-16-00722-s001.zip › cancers-2821578 - Supplementary Methods.pdf]

Supplementary Methods S1

Sequencing Methods
KAPA mRNA HyperPrep Kit

Library preparation

Total RNA quantification and integrity was confirmed using Agilent's 2100 Tapestation (Standard Total RNA assay). RINs values were confirmed to all be > 7.0, indicating high integrity RNA suitable for library prep.

For each sample, 250ng of total RNA were processed using the KAPA mRNA HyperPrep Kit (Roche p/n KK8580) according to manufacturer's instructions. Briefly, mRNA was isolated from total RNA by use of paramagnetic Oligo dT beads to pull down poly-adenylated transcripts. The purified mRNA was fragmented using chemical hydrolysis (heat and divalent metal cation) and primed with random hexamers. Strand-specific first strand cDNA was generated using Reverse Transcriptase in the presence of Actinomycin D. This allows for RNA dependent synthesis while preventing spurious DNA-dependent synthesis. The second cDNA strand was synthesised using dUTP in place of dTTP, to mark the second strand. The resultant cDNA is then "A-tailed" at the 3' end to prevent self-ligation and adapter dimerisation.

Full partial length, universal adaptors containing a T overhang are ligated to the A-Tailed cDNA. Successfully ligated cDNA molecules were then enriched and indexed with limited cycle PCR (13 PCR cycles). The high-fidelity polymerase employed in the PCR is unable to extend through uracil. This means only the first strand cDNA is amplified for sequencing, making the library strand specific (first-strand).

Sequencing

High yield, adaptor-dimer free libraries were confirmed on the Agilent TapeStation 2100 (High Sensitivity Agilent DNA 1000 assay).
Samples were quantified using the Qubit High Sensitivity DNA assay and normalised to 10nM. An equal volume of each library were pooled together and re-quantified by Qubit. Samples were sequenced on the NextSeq 500 instrument (Illumina, San Diego, US) at 1.8pM, using a 43bp paired-read run with corresponding 6bp sample index read.

Data Analysis

Run data were demultiplexed and converted to fastq files using Illumina's bcl2fastq Software v1.8. Fastq files were then aligned to the human genome UCSC hg38 using RNA-STAR 2.5.2b. Reads per transcript were counted by FeatureCounts in order to produce a digital output of gene expression. Differential expression was estimated using the BioConductor package SARTools, a DESeq2 wrapper.

All annotation and sequences were obtained from Illumina iGenomes (http://emea.support.illumina.com/sequencing/sequencing_software/igenome.html)
