# Supplementary material for: A Potential Prognostic Gene Signature Associated with p53-Dependent NTRK1 Activation and Increased Survival of Neuroblastoma Patients
Source: Cancers (Basel). 2024 Feb 8;16(4):722. doi: 10.3390/cancers16040722 (PMC10886603; doi:10.3390/cancers16040722)
Supplement: Supplementary file 1 [file cancers-16-00722-s001.zip › resubmitted supplementary table.pdf]

Table S1. List of 167 genes identified through analysis with Dseq2, EdgeR and limma-voom software packages.

	Gene name	Ensembl ID
1	INPP5F	ENSG00000198825
2	AC011330.1	ENSG00000249839
3	LINC02495	ENSG00000249896
4	ZFP3	ENSG00000180787
5	SIX1	ENSG00000126778
6	LRRC4B	ENSG00000131409
7	OSTF1	ENSG00000134996
8	GUCA1A	ENSG00000048545
9	MTMR11	ENSG00000014914
10	DLG2	ENSG00000150672
11	NUDT11	ENSG00000196368
12	ESRP1	ENSG00000104413
13	ZFR2	ENSG00000105278
14	BASP1	ENSG00000176788
15	ZNF497	ENSG00000174586
16	PHACTR1	ENSG00000112137
17	ZC3H10	ENSG00000135482
18	FOXE1	ENSG00000178919
19	NMRK1	ENSG00000106733
20	GABRB3	ENSG00000166206
21	VAT1L	ENSG00000171724
22	SLC6A16	ENSG00000063127
23	ZNF732	ENSG00000186777
24	TXNDC5	ENSG00000239264
25	MAP7D2	ENSG00000184368
26	ISY1-RAB43	ENSG00000261796
27	NKAPP1	ENSG00000233382
28	LINC01833	ENSG00000259439
29	DSC3	ENSG00000134762
30	TRIM5	ENSG00000132256
31	CYTOR	ENSG00000222041
32	HYPK	ENSG00000242028
33	NUDT10	ENSG00000122824
34	FRMD3	ENSG00000172159
35	AC020928.1	ENSG00000267260
36	B3GAT1	ENSG00000109956
37	NETO2	ENSG00000171208
38	RGS10	ENSG00000148908
39	PCDHB14	ENSG00000120327
40	TNNT1	ENSG00000105048
41	NXT2	ENSG00000101888
42	CCDC125	ENSG00000183323
43	ZNF229	ENSG00000278318
44	ZNF423	ENSG00000102935
45	NEK6	ENSG00000119408
46	TMEM178B	ENSG00000261115
47	MT-TM	ENSG00000210112
48	CNKSR3	ENSG00000153721
49	BCAN	ENSG00000132692
50	AL022311.1	ENSG00000279738
51	DENND1C	ENSG00000205744
52	SH2D3C	ENSG00000095370

53	PCDHA4	ENSG00000204967
54	PLPP3	ENSG00000162407
55	PCDHB2	ENSG00000112852
56	SIX3	ENSG00000138083
57	AC087190.3	ENSG00000263244
58	RIPPLY2	ENSG00000203877
59	IL11RA	ENSG00000137070
60	FRAS1	ENSG00000138759
61	HEBP2	ENSG00000051620
62	AGTR1	ENSG00000144891
63	FNBP1	ENSG00000187239
64	IQCE	ENSG00000106012
65	SVIL	ENSG00000197321
66	WDR6	ENSG00000178252
67	RACK1	ENSG00000204628
68	AC002351.1	ENSG00000258240
69	VWA7	ENSG00000204396
70	ITGA11	ENSG00000137809
71	IGSF10	ENSG00000152580
72	CHODL	ENSG00000154645
73	CACNA1D	ENSG00000157388
74	RPSAP48	ENSG00000240590
75	IL20RA	ENSG00000016402
76	CHRDL1	ENSG00000101938
77	SYT3	ENSG00000213023
78	PTPRK	ENSG00000152894
79	HSPA2	ENSG00000126803
80	PLPP2	ENSG00000141934
81	DEPDC4	ENSG00000166153
82	CLDN23	ENSG00000253958
83	PALM2AKAP2	ENSG00000157654
84	GUSBP5	ENSG00000236296
85	KCNQ3	ENSG00000184156
86	SHANK1	ENSG00000161681
87	AL139011.2	ENSG00000258465
88	PLXDC1	ENSG00000161381
89	DIPK2A	ENSG00000181744
90	DNM1P46	ENSG00000182397
91	SLC7A6	ENSG00000103064
92	FLVCR1	ENSG00000162769
93	CYP3A5	ENSG00000106258
94	FGF23	ENSG00000118972
95	SLITRK5	ENSG00000165300
96	LINC01363	ENSG00000231605
97	MAP3K4	ENSG00000085511
98	HRH3	ENSG00000101180
99	CD38	ENSG00000004468
100	DIO3	ENSG00000197406
101	AL133461.1	ENSG00000282413
102	FAM220A	ENSG00000178397
103	AL159163.1	ENSG00000249141
104	RNU6-33P	ENSG00000207524
105	HAPLN4	ENSG00000187664
106	ARL2-SNX15	ENSG00000273003
107	AC002456.1	ENSG00000223969
108	PROSER2	ENSG00000148426

109	SLC7A8	ENSG00000092068
110	RYR1	ENSG00000196218
111	CEBPD	ENSG00000221869
112	SLC22A4	ENSG00000197208
113	P2RY1	ENSG00000169860
114	SPECC1L-ADORA2A	ENSG00000258555
115	NR4A3	ENSG00000119508
116	PAPPA	ENSG00000182752
117	SPOCK1	ENSG00000152377
118	CD9	ENSG00000010278
119	EFNA2	ENSG00000099617
120	FRMD6	ENSG00000139926
121	THSD7A	ENSG00000005108
122	TMOD1	ENSG00000136842
123	SPG7	ENSG00000197912
124	CFAP251	ENSG00000158023
125	HSD17B12	ENSG00000149084
126	PSG2	ENSG00000242221
127	SLITRK4	ENSG00000179542
128	ITGB5	ENSG00000082781
129	TNFSF13B	ENSG00000102524
130	TDRP	ENSG00000180190
131	COL18A1	ENSG00000182871
132	WASH7P	ENSG00000227232
133	COL5A1	ENSG00000130635
134	CAVIN1	ENSG00000177469
135	PIEZO1	ENSG00000103335
136	IGF2BP1	ENSG00000159217
137	TOX2	ENSG00000124191
138	PKD1P5	ENSG00000254681
139	DUSP9	ENSG00000130829
140	AC233968.1	ENSG00000274615
141	C1QTNF3-AMACR	ENSG00000273294
142	RB1-DT	ENSG00000231473
143	SNORC	ENSG00000182600
144	RPS26P19	ENSG00000244563
145	AC048338.1	ENSG00000256861
146	AL589666.1	ENSG00000271793
147	DUOX1	ENSG00000137857
148	CHST5	ENSG00000135702
149	AC092447.7	ENSG00000237268
150	AC018809.3	ENSG00000288550
151	AL357673.1	ENSG00000256407
152	AC005670.2	ENSG00000262633
153	BCRP3	ENSG00000215481
154	ISG15	ENSG00000187608
155	IL4R	ENSG00000077238
156	AC108488.1	ENSG00000242282
157	AC023490.4	ENSG00000278044
158	AC073111.3	ENSG00000284048
159	LINC02820	ENSG00000258815
160	FCF1P2	ENSG00000228638
161	DNAAF4-CCPG1	ENSG00000261771
162	AL353691.2	ENSG00000237875
163	AC006064.6	ENSG00000285238
164	PPIA	ENSG00000196262

165	PPIB	ENSG00000166794
166	ACE2	ENSG00000130234
167	CD147	ENSG00000172270

Table S2. Clinical characteristics in data sets.

	**GSE49711/ GSE62564**	**GSE85047**	**TARGET**
No. of patients	493	272	244
% male	58.0%	N/A	57.0%
% samples diagnosed at < 18 months	61.3%	52.0%	13.0%
% samples with 5 year EFS	44.6%	79.0%	35.6%
% samples with *MYCN* amplification	18.7%	18.7%	29.1%
% samples with unfavourable tumours	18.3%	N/A	74.1%
% samples with NTRK1 pathway activation	28.4%	N/A	22.7%
% samples with INSS stage 1 tumours	24.0%	17.0%	12.0%
% samples with INSS stage 2 tumours	16.0%	13.0%	0.0%
% samples with INSS stage 3 tumours	13.0%	15.0%	0.0%
% samples with INSS stage 4 tumours	37.0%	45.0%	87.0%
% samples with INSS stage 4S tumours	11.0%	10.0%	0.0%

Table S3. Independent permutation scores of genes showing their contribution to the model.

**Genes**	**Permutation Importance**
BSG/ CD147	3.99
TOX2	3.14
HSD17B12	2.83
BASP1	2.18
FNBP1	2.14
HAPLN4	2.03

DLG2	1.96
IGSF10	1.76
CD9	1.67
KCNQ3	1.57
HEBP2	1.07
GNB2L1 / RACK1	1.02
CCDC125	0.82
GABRB3	0.74
IQCE	0.67
IL11RA	0.35
FRMD3	0.13
